# Image Classification and Recognition of Rice Diseases: A Hybrid DBN and Particle Swarm Optimization Algorithm

**DOI:** 10.3389/fbioe.2022.855667

**Published:** 2022-04-27

**Authors:** Yang Lu, Jiaojiao Du, Pengfei Liu, Yong Zhang, Zhiqiang Hao

**Affiliations:** ^1^ College of Information and Electrical Engineering, Heilongjiang Bayi Agricultural University, Daqing, China; ^2^ School of Physics and Electronic Engineering, Northeast Petroleum University, Daqing, China; ^3^ Key Laboratory for Metallurgical Equipment and Control of Ministry of Education, Wuhan University of Science and Technology, Wuhan, China

**Keywords:** image classification, image recognition, rice diseases, deep belief networks, switching particle swarm optimization algorithm

## Abstract

Rice blast, rice sheath blight, and rice brown spot have become the most popular diseases in the cold areas of northern China. In order to further improve the accuracy and efficiency of rice disease diagnosis, a framework for automatic classification and recognition of rice diseases is proposed in this study. First, we constructed a training and testing data set including 1,500 images of rice blast, 1,500 images of rice sheath blight, and 1,500 images of rice brown spot, and 1,100 healthy images were collected from the rice experimental field. Second, the deep belief network (DBN) model is designed to include 15 hidden restricted Boltzmann machine layers and a support vector machine (SVM) optimized with switching particle swarm (SPSO). It is noted that the developed DBN and SPSO-SVM can simultaneously learn three proposed features including color, texture, and shape to recognize the disease type from the region of interest obtained by preprocessing the disease images. The proposed model leads to a hit rate of 91.37%, accuracy of 94.03%, and a false measurement rate of 8.63%, with the 10-fold cross-validation strategy. The value of the area under the receiver operating characteristic curve (AUC) is 0.97, whose accuracy is much higher than that of the conventional machine learning model. The simulation results show that the DBN and SPSO-SVM models can effectively extract the image features of rice diseases during recognition, and have good anti-interference and robustness.

## 1 Introduction

Rice is one of the most important food crops for our people. In recent years, the yield of the rice planting industry has increased day by day, but there are still some adverse factors affecting its yield, such as rice diseases. At present, rice blast, rice sheath blight, and rice brown spots have become the most common diseases in the cold areas of northern China. It can occur during the whole growth period of rice, which has a great impact on the yield and quality of rice, and even lead to no yield in serious cases. This will not only affect the income of farmers but also have a bad impact on China’s food security and fiscal revenue. At present, the identification methods of rice diseases mainly rely on farmers’ experience to judge, consult disease books or query on the Internet, consult agricultural technicians, or seek help from plant experts. However, the recognition efficiency of human eyes is low, and the recognition is highly subjective. There may be misjudgment, which makes it difficult to realize the accurate identification of diseases. The update speed of disease books is slow, the amount of information on the Internet is large, and the accuracy cannot be guaranteed. By consulting agricultural technicians and plant experts, a lot of material and financial resources will be consumed, a lot of time and cost will be spent, and it is easy to miss the best opportunity for disease treatment. Therefore, finding a new, efficient, and accurate rice disease recognition method to replace the traditional human eye recognition method is the key to solving the problem of rice disease recognition, which has important research significance.

However, so far, no research has involved the integration of the DBN and SPSO-SVM, because the DBN and SPSO-SVM have great potential application in rice disease identification. Based on the shortcomings and limitations of traditional identification methods, we proposed a method based on the DBN and SPSO-SVM to identify rice blast, sheath blight, and brown spot. Using the images in the rice disease database, the DBN and SPSO-SVM models are trained in two steps. First, a DBN model is constructed using RBMs. Second, the gradient descent algorithm is used to train the DBN and SVM optimized by SPSO as a classifier. The DBN and SPSO-SVM are then evaluated by 10-fold cross-validation.

The main innovations of this study are as follows: a novel framework for automatic diagnosis of rice diseases is given to settle the problems in image segmentation of rice disease spot, where the DBN and SPSO-SVM method are applied to accurately extract the edge of rice disease spot; by learning disease spot features, the proposed DBN and SPSO-SVM can identify the region of interest that is obtained by preprocessing the rice disease images. Experimental results show that this method has high accuracy in feature parameters, segmentation, and peak signal-to-noise ratio. This research replaces the traditional rice disease diagnosis method, enhances the in-depth integration of the rice disease identification field and machine learning technology, combines the DBN algorithm with the SPSO-SVM algorithm for rice disease identification for the first time, effectively improves the accuracy and timeliness of rice disease control, and has practical significance.

The rest of the study is given as follows: Section 2 introduces the relevant research status at home and abroad; Section 3 collects and extracts the characteristics of rice diseases; Section 4 describes the architecture of the DBN and SPSO-SVM framework; Section 5 applies the developed DBNs and SPSO-SVM to the identification of three diseases in rice and demonstrates the experimental results; and conclusions are given in Section 6.

## 2 Related Work

In recent years, with the rapid development of digital image processing, computer vision, and pattern recognition technology, new ways and methods have been provided for rapid and accurate diagnosing, analyzing, classifying, and nondestructive detection of plant diseases (see Zhang D. et al., 2018; Kaur et al., 2019; Li Y. et al., 2019; Fuentes et al., 2020; Li et al., 2020; Madhulatha and Ramadevi, 2020; Nanehkaran et al., 2020; Sethy et al., 2020; Wspanialy and Moussa, 2020; Li and Chao, 2021, and the references therein). These intelligent diagnostic methods have achieved great success in both theoretical research and practical application. However, due to the limitation of theoretical analysis and the need for specific skills and a lot of experience knowledge in the course of training, the effect of diagnosis is not optimal in many cases.

A deep belief network (DBN) has been proposed by Hinton and Salakhutdinov (2006) at the University of Toronto in 2006, and the relevant results have been reported in the study by Hinton (2012), Dong et al. (2015b), Russakovsky et al. (2015), and Dong et al. (2016b). During this period, the DBN is a probabilistic generation model, which consists of multiple restricted Botlzmann machines (RBMs). It can extract features from the original data by stacking the RBMs layer by layer; then we can obtain some high-level representations of the original data. The core of this deep learning model is to optimize the connection weight of the deep neural network by the layer-by-layer greedy learning algorithm. First, in order to mine the disease features of the rice, unsupervised layer-by-layer training is used. Then, by adding the corresponding classifier and reversing the supervised fine-tuning, the disease diagnosis ability of the DBN is optimized.

The switching PSO algorithm has been proposed by (Tang et al., 2011), in which a mode-dependent velocity updating Eq. 7 is introduced to realize a balance between the local search algorithm and the global one. Lu et al. (2015) have employed the SPSO algorithm to solve the optimization problem with constraints by converting it into an optimization problem without constraints and have gained a better performance.

In the last few years, lots of researchers have applied the DBN in many application fields such as image recognition, speech recognition, face recognition, and plant leaf classification (Cheng et al., 2018; Goudarzi et al., 2018; Liu et al., 2018; Cristin et al., 2020; Vedantham and Reddy, 2020; Veisi and Mani, 2020; Chen and Pan, 2021). It is a multi-layer network structure, which provides a basic model for feature extraction and subsequent image recognition of original images. The DBN has become a hot research topic in the field of image recognition.

As for the identification and classification of plant leaf diseases, a convolution neural network has been introduced by some researchers. For example, Rahman and Arko et al. (Rahman et al., 2020) proposed a two-stage small CNN architecture, which effectively avoids the disadvantages that the large model architecture is not suitable for mobile devices, reduces the model size while ensuring accuracy, and is more widely used in practical production. Zhang and Qiao et al. (Zhang X. et al., 2018) improved GoogLeNet and Cifar10 models based on deep learning, which are proposed to identify corn leaf diseases. The experimental results show that the improved method reduces the number of convergence iterations and effectively improves the accuracy of corn leaf diseases. Hassan and Maji et al. (Hassan et al., 2021) have recognized plant leaf diseases using the CNN and transfer learning methods. Sharma and Berwal et al. (Sharma et al., 2020) have used the S-CNN model trained by segmented images to detect plant diseases. Hu and Yang et al. (Hu et al., 2019) have used an improved deep convolutional neural network model to identify tea leaf diseases. Atila and Uçar et al. (Atila et al., 2021) have used an efficient deep learning model classification of plant leaf diseases. Therefore, we propose to combine the DBN and SPSO-SVM for the identification of rice blast, sheath blight, and brown spot, so as to improve the identification efficiency and diagnostic accuracy.

## 3 Data Acquisition and Feature Extraction

### 3.1 Data Collection and Processing

**FIGURE 1:**
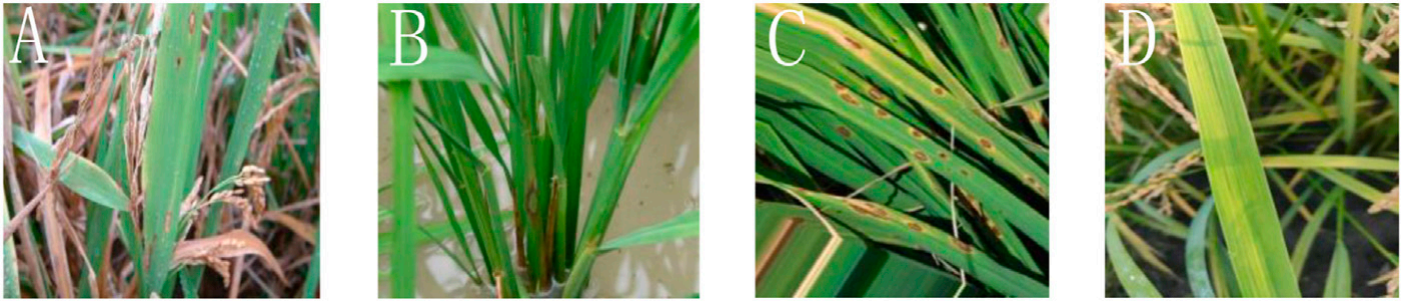
Rice diseases and health image. **(A)** Rice blast image, **(B)** sheath blight image, **(C)** brown spot image, and **(D)** healthy rice image.

**FIGURE 2:**
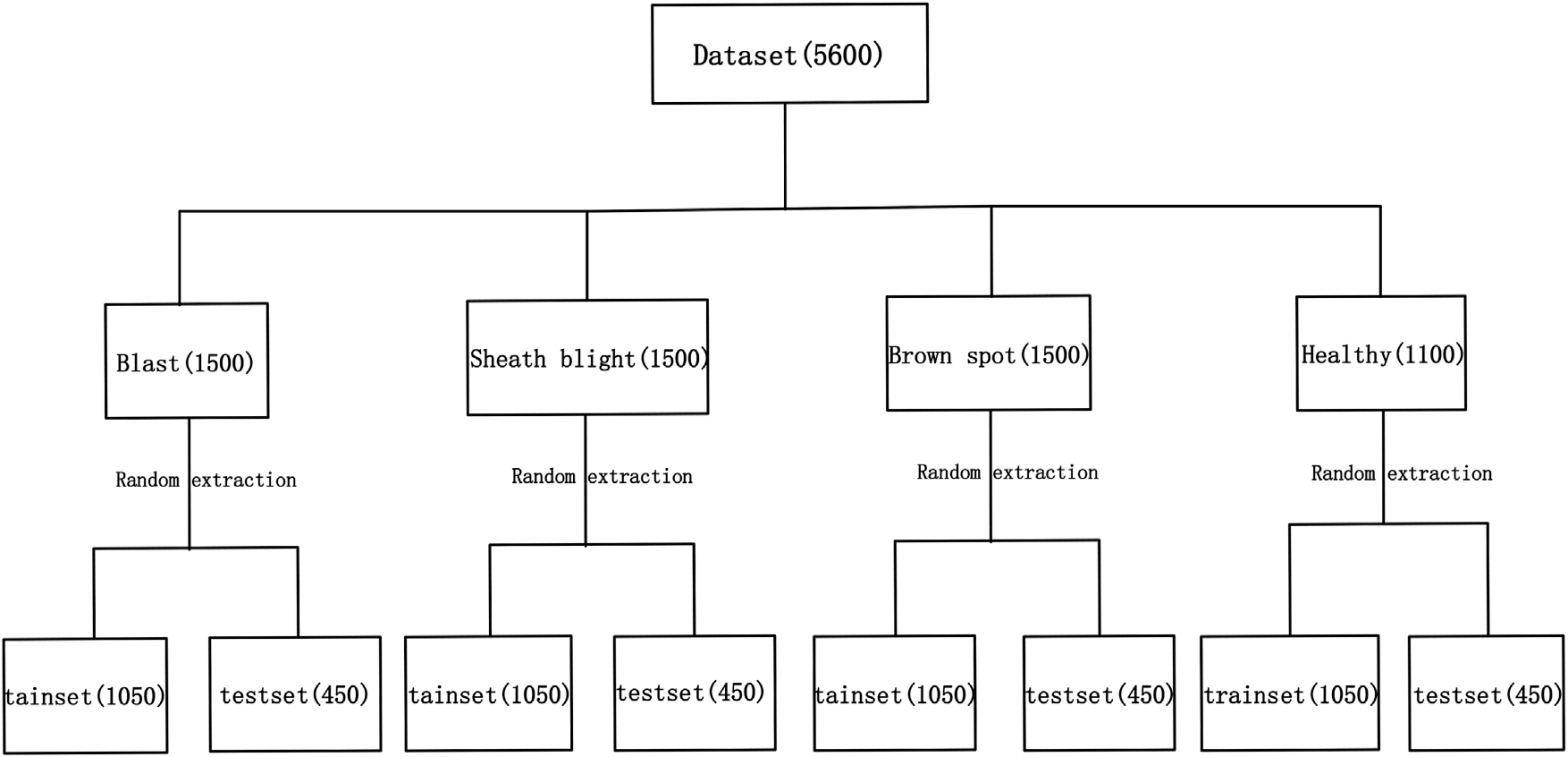
Data set partition.

Under natural light conditions, using iPhone seven Plus mobile phone (IOS 10.1 operating system, F/1.8 wide-angle, and F/2.8 telefocal dual-lens camera, 12 million pixels), the rice disease images are captured in the rice experimental field of Heilongjiang Academy of Agricultural Reclamation Sciences, China. The mobile phone is fixed on a bracket, using automatic exposure mode, taking a picture at a moderate distance from the diseased rice leaves. The photo size taken by the phone is 3,024 × 4,032 pixels. The images of three rice diseases were obtained in the early, middle, and late stages, respectively. In each cycle, 500 images were collected for each of the three rice disease images. Therefore, a total of 5,600 sample images were collected, including 1,500 images of rice blast, sheath blight, and brown spot, of which 1,100 were uninfected. Each image is stored in the PNG format and compressed into a uniform size of 400, ×, 300. An example of the original rice disease image sample is shown in Figure 1; 70% of the samples are randomly selected from the data set as a training set, and the remaining 30% of the samples are chosen as a testing set. The data set division is shown in Figure 2.

### 3.2 Feature Extraction

The feature extraction of the disease spot image is a key step in rice disease recognition. According to the spot characteristics of rice sheath blight (taking rice sheath blight as an example), three features of color, texture, and shape are extracted from the spot image.

#### 3.2.1 Color Feature Extraction

Color feature is not affected by the direction, size, and angle of the image. It is a typical visual feature in the image and is widely used in the field of image recognition. At present, some researchers have achieved good results in this field. Peihua Shi used machine learning to estimate rice nitrogen nutrition through RGB images. In this study, the color eigenvalues of rice diseases are extracted from RGB, HSV, and lab color spaces. First, the rice disease image containing disease spots is transformed from RGB color space to HSV color space, and the mean, variance, and energy of the color set in HSV space are obtained as part of the color characteristics. Then, the third-order color moment corresponding to each component in HSV, RGB, and lab space is obtained as another part of the color feature. The color moment is calculated as follows:

1) First-order color moment (mean): reflects the average brightness of the image. It is defined as

$$\mu =\frac{1}{N}\overset{N}{\underset{j=1}{\sum }}p_{i,j}.$$ (1)

2) Second-order color moment (variance): reflects the discreteness of image gray level distribution. It is defined as

$$\sigma_{i}={\left[\frac{1}{N}\overset{N}{\underset{j=1}{\sum }}{\left(p_{i,j}-\mu_{i}\right)}^{2}\right]}^{\frac{1}{2}}.$$ (2)

3) Third-order color moment: defines the skewness of color component and color asymmetry. It is defined as

$$s_{i}={\left\{\frac{1}{N}\overset{N}{\underset{j=1}{\sum }}{\left(p_{i,j}-\mu_{i}\right)}^{3}\right\}}^{\frac{1}{3}}$$ (3)

where *p*_*i*,*j*_ represents the *i*th color component of the *j*th pixel of the color image, and N represents the number of pixels in the image.

#### 3.2.2 Texture Feature Extraction

**FIGURE 3:**
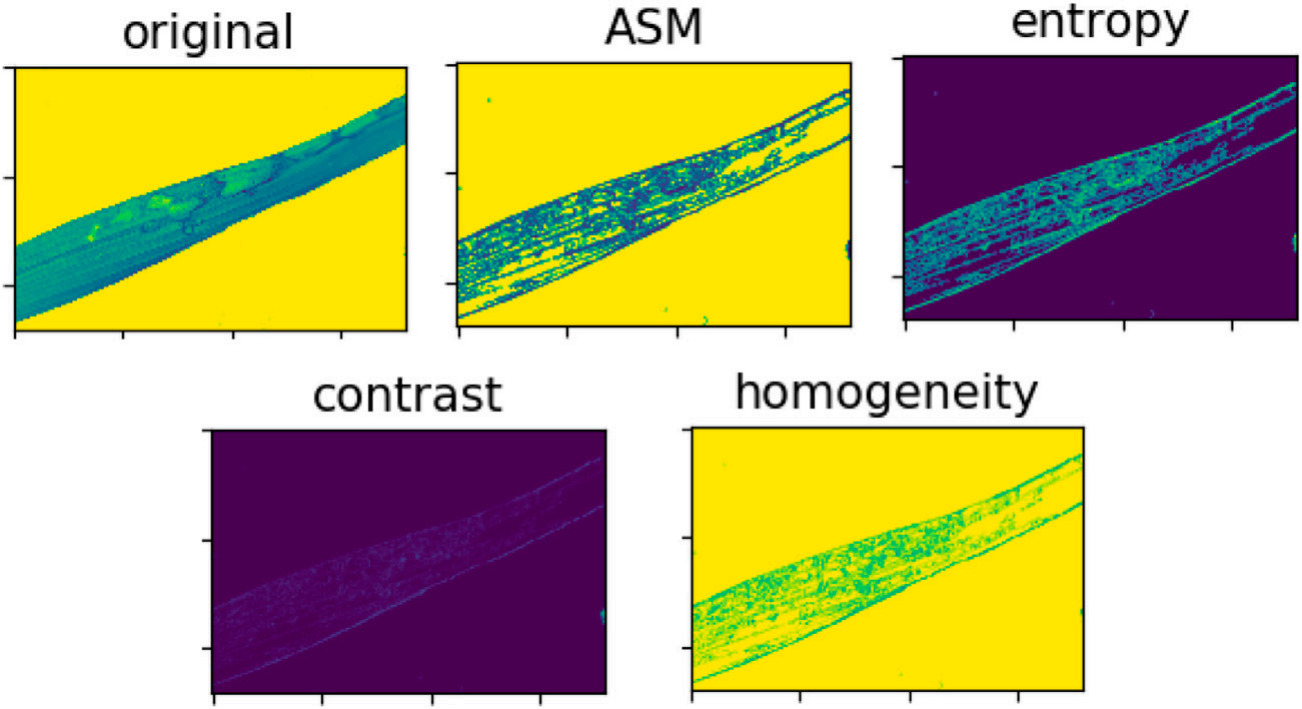
Texture feature extraction disease spot.

Texture feature can reflect the organization and arrangement of the components of things. It is an important visual feature in image description. It can calculate the statistical features of multiple pixel regions and has rotation invariance. As the most widely used texture feature extraction method, the gray level co-occurrence matrix method was proposed by Haralick et al. in 1973. It can effectively describe the change information of image in a certain distance and direction. Therefore, the gray level co-occurrence matrix is used to extract texture features in this study. The texture feature extraction effect is shown in Figure 3. The gray level co-occurrence matrix can be expressed by the following formula:

$$P\left(a_{1},a_{2}\right)=\frac{\#\left\{\left[\left(x_{1},y_{1}\right),\left(x_{2},y_{2}\right)\right]\in S|f\left(x_{1},y_{1}\right)=a_{1}\&f\left(x_{2},y_{2}\right)=a_{2}\right\}}{\#S}$$ (4)

In the formula, *f* (*x*, *y*) represents the image itself, S represents the set of pixel pairs with some spatial relationship in the image, the expression on the numerator on the right side of the equation represents the number of pixel pairs with some spatial relationship and gray value of *a*_1_, *a*_2_, and the expression on the denominator represents the number of total pixel pairs (*#* represents the number of statistical elements).

The common feature parameters extracted according to the gray level co-occurrence matrix are as follows:

1) Energy or angular second-order moment: it is a measure of the uniformity of image gray distribution and the thickness of texture.

$$ASM=\overset{N}{\underset{i=1}{\sum }}\overset{N}{\underset{j=1}{\sum }}P{\left(i,j\right)}^{2}$$ (5)

2) Entropy: it reflects the nonuniformity or complexity of image texture. It is a measure of the amount of texture information in the image.

$$ENT=-\overset{N}{\underset{i=1}{\sum }}\overset{N}{\underset{j=1}{\sum }}P\left(i,j\right)\cdot \log P\left(i,j\right)$$ (6)

3) Contrast: it reflects the clarity of the image and measures the depth of the image texture groove.

$$CON=\overset{M}{\underset{i=1}{\sum }}\overset{N}{\underset{j=1}{\sum }}{\left(i-j\right)}^{2}p\left(i,j\right)$$ (7)

4) Inverse differential moment: it reflects the clarity and regularity of image texture and the local change of image.

$$IDM=\overset{M}{\underset{i=1}{\sum }}\overset{M}{\underset{j=1}{\sum }}\frac{P\left(i,j\right)}{1+{\left(i-j\right)}^{2}}$$ (8)

#### 3.2.3 Shape Feature Extraction

In this study, on the basis of selecting the conventional morphological features, such as lesion area, lesion circularity, lesion complexity, and the number of lesions, two new morphological features, namely, the number of lesions and the ratio of lesion area to the number of lesions, are proposed. The calculation formula of lesion area s is

$$S=\overset{n}{\underset{x=1}{\sum }}\overset{m}{\underset{y=1}{\sum }}f\left(x,y\right)$$ (9)

where *f* (*x*, *y*) is a binary image function. Circularity C represents the deviation degree between the shape of the lesion area and the circle, and the calculation formula is

$$C=d_{\max}-d_{\min}$$ (10)

where *d*_max_ − *d*_min_ is the maximum/minimum diameter of the cross section of the lesion. Lesion complexity E describes the perimeter per unit area of the lesion area, and the calculation formula is

$$E=\frac{4\Pi }{C}$$ (11)

Number of disease spots N represents the number of rice disease spots on a diseased leaf. The ratio of lesion area to lesion number is

$$R=\frac{S}{N}$$ (12)

**FIGURE 4:**
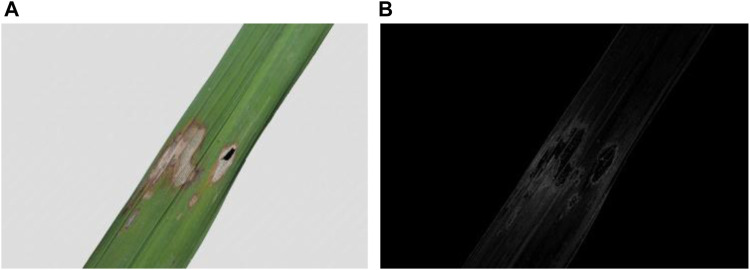
Shape feature extraction of rice disease spot.

The effect of rice disease feature extraction is shown in Figure 4.

## 4 Architecture of DBN and SPSO-SVM Framework

### 4.1 Restricted Boltzmann Machine

**FIGURE 5:**
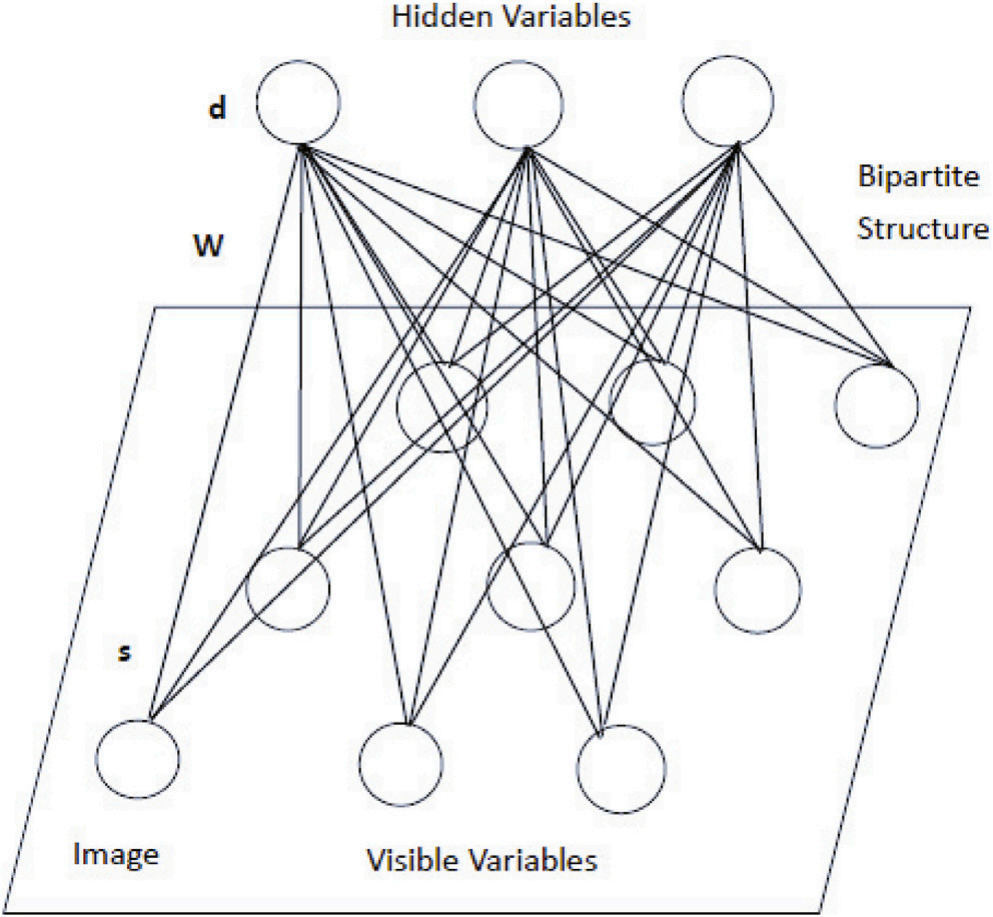
Schematic diagram of the RBM.

In Figure 5, an energy model called restricted Boltzmann machine (RBM) is shown. It is a special kind of stochastic neural network model that owns a two-layer architecture. The input layer is also called the visible layer, in which visible units represent observations. The other layer is called the hidden layer, in which hidden units represent features. Visible variables *S* are linked to hidden variables *D* through undirected weighted connections *W*. There are no connections in the same layer. This constitutes a bipartite structure graph. The parameters are introduced as follows:

$S=\left(s_{1},s_{2},\ldots ,s_{m}\right)\in R^{m}$ , where *m* is the neurons’ number in the visible layer.

$D=\left(d_{1},d_{2},\ldots ,d_{n}\right)\in R^{n}$ , where *n* is the neurons’ number in the hidden layer.

$A=\left(a_{1},a_{2},\ldots ,a_{m}\right)\in R^{m}$ represents bias in the visible layer.

$B=\left(b_{1},b_{2},\ldots ,b_{n}\right)\in R^{n}$ represents bias in the hidden layer.

$W=\left(w_{i,j}\right)\in R^{n\times m}$ stands for the weight matrix that connects the visible layer and the hidden layer. *w*_*i*,*j*_ is the weight that connects the *jth* neuron in the hidden layer and the *ith* neuron in the visible layer.

*ϱ* = (*W*, *A*, *B*) represents the unknown model parameter.

RBM is an energy based model. Assuming that the variables of both visible and hidden layers obey Bernoulli distribution, the joint probability distribution of visible and hidden variables is given by energy function. For a given state (*s*, *d*), an energy function can be defined as follows (Hinton and Salakhutdinov, 2006):

$$E_{\varrho }\left(s,d\right)=-\overset{m}{\underset{i=1}{\sum }}a_{i}s_{i}-\overset{n}{\underset{j=1}{\sum }}b_{j}d_{j}-\overset{m}{\underset{i=1}{\sum }}\overset{n}{\underset{j_{1}}{\sum }}d_{j}w_{j,i}s_{i}.$$ (13)

Then, the joint probability distribution of *s*, *d* is given as follows:

$$P_{\varrho }\left(s,d\right)=\frac{1}{Z_{\varrho }}e^{-E_{\varrho }\left(s,d\right)}$$ (14)

where $Z_{\varrho }=\sum_{s,d}e^{-E_{\varrho }\left(s,d\right)}$ represents the normalization factor. Afterward, the marginal probabilities with which the model assigns to a visible vector *s* and a hidden vector *d* are given by

$$P_{\varrho }\left(s\right)=\underset{d}{\sum }P_{\varrho }\left(s,d\right)=\frac{1}{Z_{\varrho }}\underset{d}{\sum }e^{-E_{\varrho }\left(s,d\right)}$$ (15)

$$P_{\varrho }\left(d\right)=\underset{s}{\sum }P_{\varrho }\left(s,d\right)=\frac{1}{Z_{\varrho }}\underset{s}{\sum }e^{-E_{\varrho }\left(s,d\right)}.$$ (16)

Combined with the joint probability distribution, marginal probability distribution, and *sigmoid* function, we can get the probability of activation of a neuron on the hidden layer when given the visible layer state is given as follows:

$$P\left(d_{\tau }=1|s\right)=sigmoid\left(b_{\tau }+\overset{m}{\underset{i=1}{\sum }}w_{\tau ,i}s_{i}\right)$$ (17)

The probability of activation of a neuron on the visible layer when given the hidden layer state is given as follows:

$$P\left(s_{\tau }=1|d\right)=sigmoid\left(a_{\tau }+\overset{n}{\underset{i=1}{\sum }}w_{j,\tau }d_{j}\right)$$ (18)

where $sigmoid\left(x\right)={\left(1+e^{-x}\right)}^{-1}$ .

Given training set *X* = *s* ^1^, *s* ^2^, … , *s* ^*l*^, $S^{i}={\left(s_{1}^{i},s_{2}^{i},\ldots ,s_{m}^{i}\right)}^{T}$ , where *l* is the number of training samples. Training the RBM is to adjust the parameters *ϱ* = (*w*, *a*, *b*) so that the probability distribution represented by the RBM is consistent with the training data as far as possible.

The parameters *ϱ* = (*w*, *a*, *b*) can be solved by maximizing the likelihood function of visual layer data as follows:

$$P_{\varrho }\left(s\right)=\frac{1}{Z_{\varrho }}\underset{d}{\sum }\exp\left(s^{T}wd+a^{T}d+b^{T}s\right).$$ (19)

Considering the contrastive divergence (CD) algorithm proposed by Hinton (Hinton, 2002), the conditional probability distribution of the hidden layer and visual layer is given as follows:

$$P\left(d|s\right)=\underset{j}{\prod }P\left(d_{j}|s\right),P\left(d_{j}=1|s\right)=\frac{1}{1+\exp\left(\sum_{i}w_{ij}s_{i}-a_{j}\right)}$$ (20)

$$P\left(s|d\right)=\underset{i}{\prod }P\left(s_{i}|d\right),P\left(s_{i}=1|d\right)=\frac{1}{1+\exp\left(\sum_{j}w_{ij}d_{j}-b_{i}\right)}.$$ (21)

The updating rules for the parameters are given as follows:

$$\Delta w_{ij}=\epsilon \left({\langle s_{i}d_{j}\rangle }_{data}-{\langle s_{i}d_{j}\rangle }_{recon}\right)$$ (22)

$$\Delta a_{i}=\epsilon \left({\langle s_{i}\rangle }_{data}-{\langle s_{i}\rangle }_{recon}\right)$$ (23)

$$\Delta b_{j}=\epsilon \left({\langle d_{j}\rangle }_{data}-{\langle d_{j}\rangle }_{recon}\right)$$ (24)

where Δ is the learning rate; Δ*w*_*ij*_, Δ*a*_*i*_, and Δ*b*_*j*_ are the updating values of the weight and biases in the visible layer and hidden layer, respectively; ⟨⋅⟩_*data*_ means the values of visible variable *i* multiplied by hidden variable *j* before reconstruction; and ⟨⋅⟩_*recon*_ means the values after reconstruction. ⟨⋅⟩_*data*_ and ⟨⋅⟩_*recon*_ represent the distribution defined by the reconstruction model.

Reconstructed visible variable *s*′ and hidden variable *d*′ are one sampling of the original variable *p* (*s*, *d*); therefore, the sample set obtained by multiple sampling can be regarded as an approximation of the original variables.

### 4.2 Deep Belief Network

The typical deep belief network (DBN) structure was proposed by Hinton and Salakhutdinov (2006). It is a deep learning neural network which consists of three RBM layers and one back propagation (BP) layer. The advantage of the DBN is its capability of extracting nonlinear features by hidden layer units through unlabeled training data in visible layer units. Training a DBN contains two steps. The first step is pretraining: weights of the generative model are obtained through unsupervised greedy layer-by-layer pretraining. The second step is fine-tuning, which improves the generalization ability of the discriminant model using the gradient descent algorithm.

According to the contrastive divergence (CD) algorithm, in the pretraining step, the weights and the bias of DBN are updated by

$$w=w+\Delta \left[p\left(d^{\left(0\right)}=1|s^{\left(0\right)}\right)s^{\left(0\right)T}-p\left(d^{\left(1\right)}=1|s^{\left(1\right)}\right)s^{\left(1\right)T}\right]$$ (25)

$$b=b+\Delta \left[p\left(d^{\left(0\right)}=1|s^{\left(0\right)}\right)-p\left(d^{\left(1\right)}=1|s^{\left(1\right)}\right)\right]$$ (26)

$$a=a+\Delta \left[s^{\left(0\right)}-s^{\left(1\right)}\right]$$ (27)

where *p* (*d* ^(0)^ = 1|*s* ^(0)^) = *σ*(*w*_*j*_ ⋅ *s* ^(0)^ + *b*_*j*_) is the probability of the neurons in the hidden layer that will be turned on for Bayesian visible neurons, and Δ is the learning rate.

In the fine-tuning step, for the output BP layer, the minimum mean square error (MSE) criterion is used, and the cost function is as follows:

$$E=\frac{1}{N}\overset{N}{\underset{i=1}{\sum }}{\left({\hat{X}}_{i}\left(w^{l},b^{l}\right)-X_{i}\right)}^{2}$$ (28)

where *E* is the MSE of DBN, ${\hat{X}}_{i}$ and *X*_*i*_ represent the expected output and the actual output, respectively, *i* is the sample index, and (*w* ^*l*^, *b* ^*l*^) represent the weights and bias parameters to be learned in the *lth* layer.

The gradient descent method is used to update the weights and bias parameters of the network according to

$$\left(w^{l},b^{l}\right)=\left(w^{l},b^{l}\right)+\Delta \cdot \frac{\partial E}{\partial \left(w^{l},b^{l}\right)}.$$ (29)

### 4.3 Switching PSO Algorithm

To realize a balance between the local and the global search, a velocity update equation that depends on the mode with Markov parameters is introduced in the PSO algorithm. In general, the particles in the early stage should maintain their independence and diversity, which is helpful to expand the search area and avert premature convergence to local optimum. In the end, all groups may converge to the best particles and obtain a more accurate solution. The evolutions of the particles’ velocity and position are shown as

$$\begin{array}{cc} s_{i}\left(\tau +1\right) & =w\left(\xi \left(\tau \right)\right)s_{i}\left(\tau \right)+c_{1}\left(\xi \left(\tau \right)\right)\gamma_{1}\left(p_{1}\left(\tau \right)-x_{i}\left(\tau \right)\right)+c_{2}\left(\xi \left(\tau \right)\right)\gamma_{2}\left(p_{g}\left(\tau \right)-x_{i}\left(\tau \right)\right)\\ x_{i}\left(\tau +1\right) & =x_{i}\left(\tau \right)+s_{i}\left(\tau +1\right) \end{array}$$ (30)

where *w* (*ξ*(*τ*)), *c*_1_ (*ξ*(*τ*)), and *c*_2_ (*ξ*(*τ*)) are the inertia weight and acceleration coefficients. They all depend on a Markovian chain. The Markov chain *ξ*(*τ*) takes values in a finite state space $R=\left\{1,2,\ldots ,N\right\}$ with probability transition matrix $\Pi^{\tau }={\left(\pi_{ij}^{\left(\tau \right)}\right)}_{N\times N}$ . $\pi_{ij}^{\tau }\geq 0\left(i,j\in R\right)$ stands for the transition probability from *i* to *j* satisfying $\sum_{j=1}^{N}\Pi_{ij}^{\tau }=1$ . According to the current search information, Π^*τ*^ can be adjusted by

$$Div\left(k\right)=\frac{1}{R\cdot |L|}\overset{R}{\underset{i=1}{\sum }}\sqrt{\overset{O}{\underset{\iota =1}{\sum }}\left(x_{i\iota }\left(\tau \right)\right)-{\bar{x}}_{\iota }{\left(\tau \right)}^{2}}$$ (31)

so as to balance the global and local search abilities, where *R* represents the size of the swarm. The length of the longest diagonal in the search space is denoted by ∣*L*∣. The dimension of the object problem is *O*. *x*_*iι*_ is the *ι*th value of the *i*th particle, and the *ι*th value of the average point $\bar{x}$ is represented by ${\bar{x}}_{\iota }$ , which is shown as

$${\bar{x}}_{\iota }=\frac{1}{R}\overset{R}{\underset{i=1}{\sum }}x_{i\iota }\left(\tau \right)$$ (32)

In the study, the BP layer of DBN is replaced by the SPSO-SVM classifier (For detail of switching PSO to optimize the parameters of SVM, refer to the study by Lu et al. (2015).

### 4.4 Image Processing of Rice Diseases Based on DBN and SPSO-SVM Framework

**FIGURE 6:**
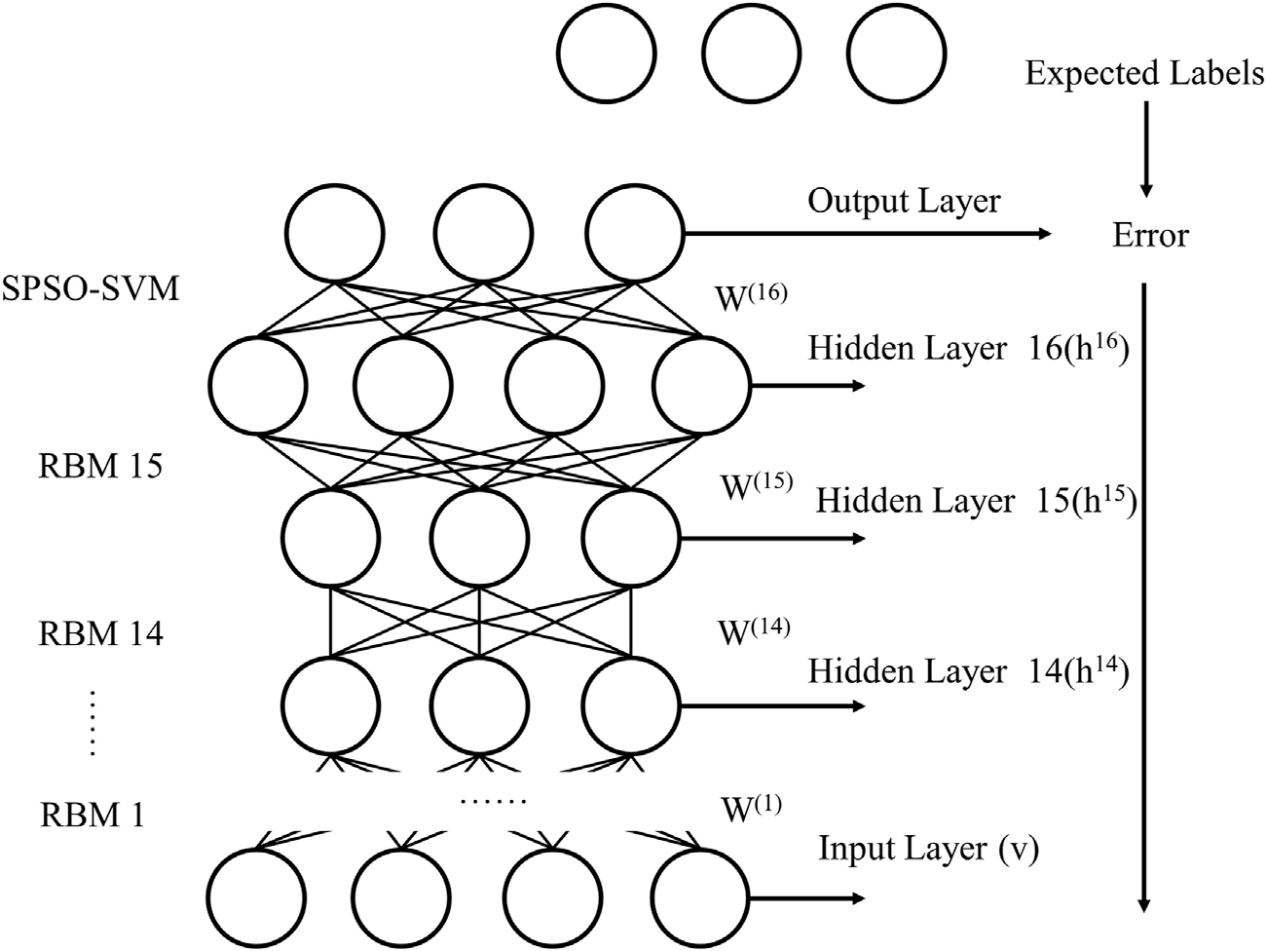
Schematic diagram of the DBN and SPSO-SVM.

This section introduces rice disease identification based on the DBN and SPSO-SVM framework. The top layer of the traditional DBN model is the BP neural network used to complete the prediction. In this study, the hybrid kernel support vector machine method optimized by the self-adaptive particle swarm optimization (SPSO) algorithm based on particle swarm concentration is used to replace the BP neural network, Combined with the DBN algorithm to detect rice diseases, the improved DBN model is shown in Figure 6.

**FIGURE 7:**
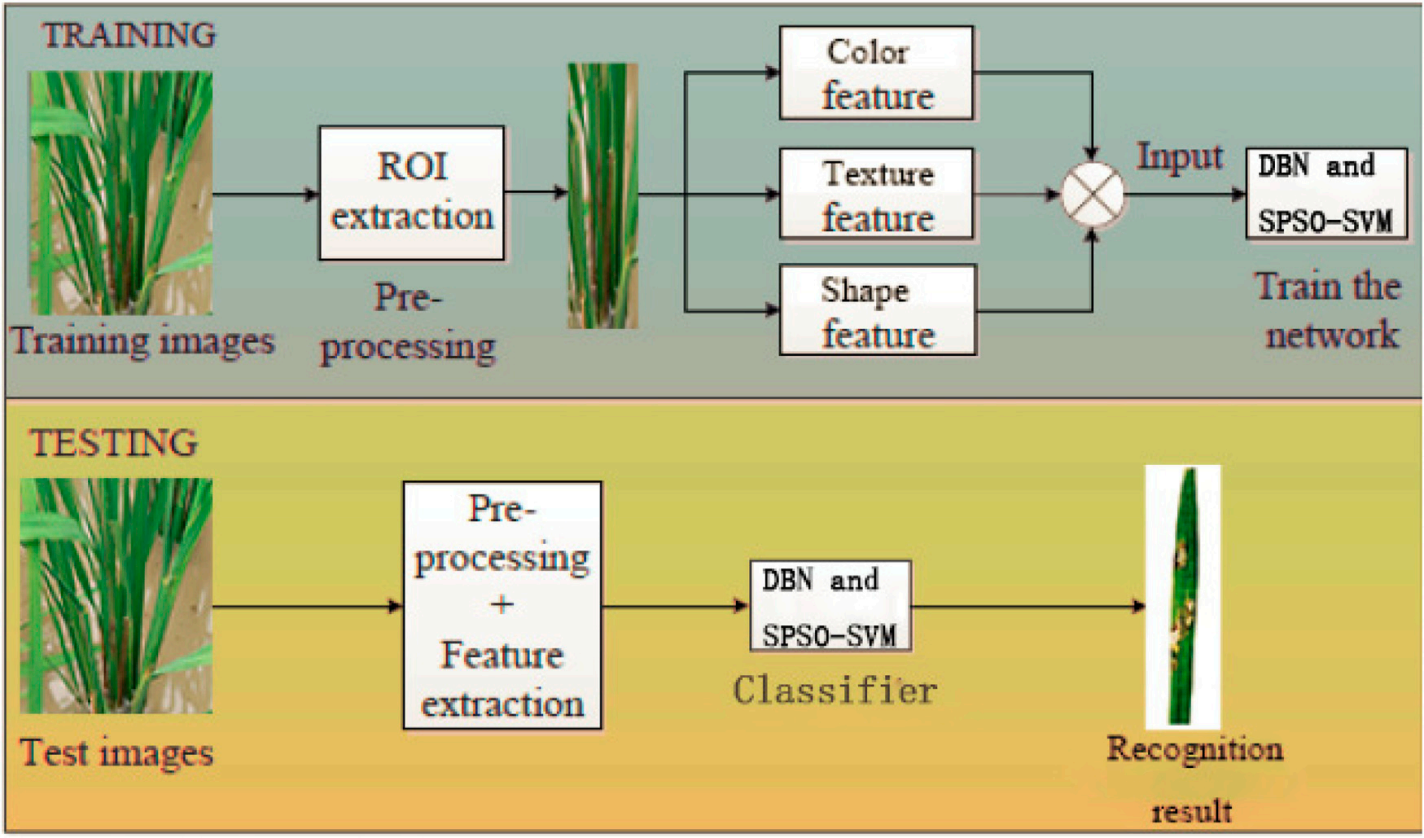
Flowchart of the DBN and SPSO-SVM-based rice diseases image recognition.

This section introduces the process of rice diseases image *via* the DBN and SPSO-SVM framework. The flowchart of rice diseases image recognition based on the DBN and SPSO-SVM is shown in Figure 7, and our aim is to learn features to identify the rice diseases. To reduce the computational complexity in the process of feature extraction by the DBN, we extract the region of interest (ROI) containing the lesion. The ROI is extracted by combining interactive and automatic extraction methods. First, the experienced plant protection experts mark the outline of the largest section of the lesion in the rice disease images. Then the maximum outer rectangular frame is constructed with the largest section. Finally, the rectangular frame is moved up, down, left, and right by five pixels to obtain the final ROI frame. We adjust the extracted ROI to a uniform scale of 64 × 64 to compare the discriminatory ability of different images against rice disease, and to adjust the network structure and parameter to analyze the impact of classification diagnosis results.

The selection of the input feature is very important for the DBN because it plays a decisive role in the performance of identification. In this study, the color feature, the shape feature, and the texture feature are selected as the input features of the DBN. The color feature is selected because infected rice tissue usually changes its color from green to brown to yellow. The texture feature is selected to detect the rice disease as there are great differences in thickness and direction between healthy and diseased tissues. Also, the shape feature can better capture the edge and internal pixel features of the lesion target.

In the pretraining stage, after three features are input into the visible layer of the first RBM, each layer of the DBN network is trained independently by an unsupervised algorithm, then we obtain the weight of the generated model. In the fine-tuning stage, the SPSO-SVM is proposed to take the output eigenvector of RBM as its input eigenvector and then train the SPSO-SVM classifier using the supervised algorithm. Moreover, in order to ensure the whole DBN eigenvector mapping is optimal, the backpropagation network will also forecast the error information from back to front to each layer, and then the weights of the DBN are fine-tuned. Moreover, for the sake of avoiding falling into the local optimum of the SVM, the SPSO has been introduced.

## 5 Rice Disease Identification Experiment

### 5.1 Test Platform and Training Parameters

The images are processed and the algorithm is implemented by using the Python 3.6 and TensorFlow 2.0 + Keras 2.2.4 open-source framework for deep learning with the Ununtu 18.04 X64 operating system. The computer has 64 GB memory and Intel (R) i7 5960K CPU at3.5 GHz processor. NVIDIA Titan XP graphics card is used to improve the image processing speed.

**FIGURE 8:**
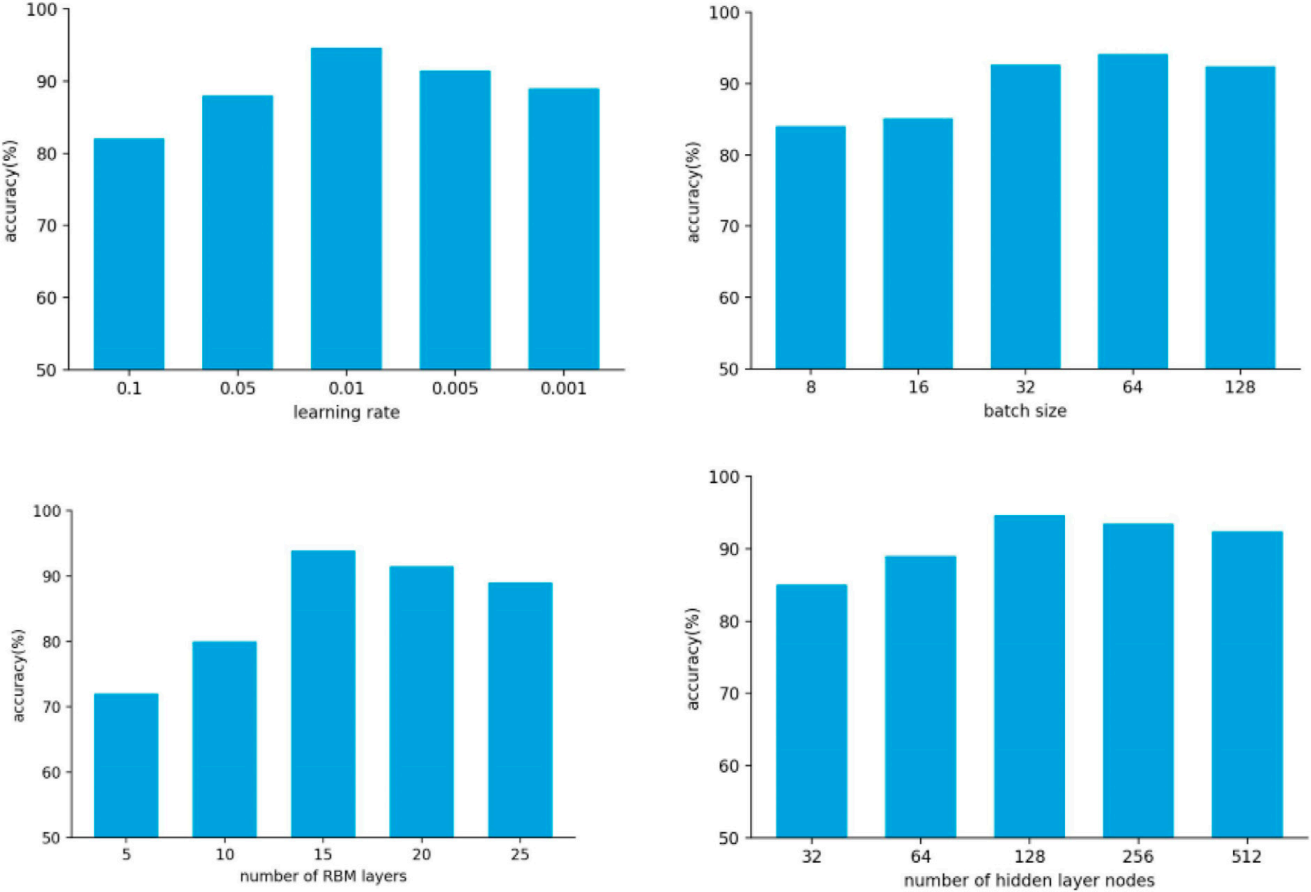
Model parameter setting.

The identification of rice blast, sheath blight, and brown spot based on the DBN and SPSO-SVM mainly refers to the influence on the accuracy. After experimental comparison, it was finally determined that the number of RBM hidden layers was 15, the number of hidden layer nodes was 128, the batch size was 64, the learning rate was 0.01, the optimizer was SGD, and the momentum was 0.9. The comparison of test results is shown in Figure 8.

### 5.2 Experiment Results

**FIGURE 9:**
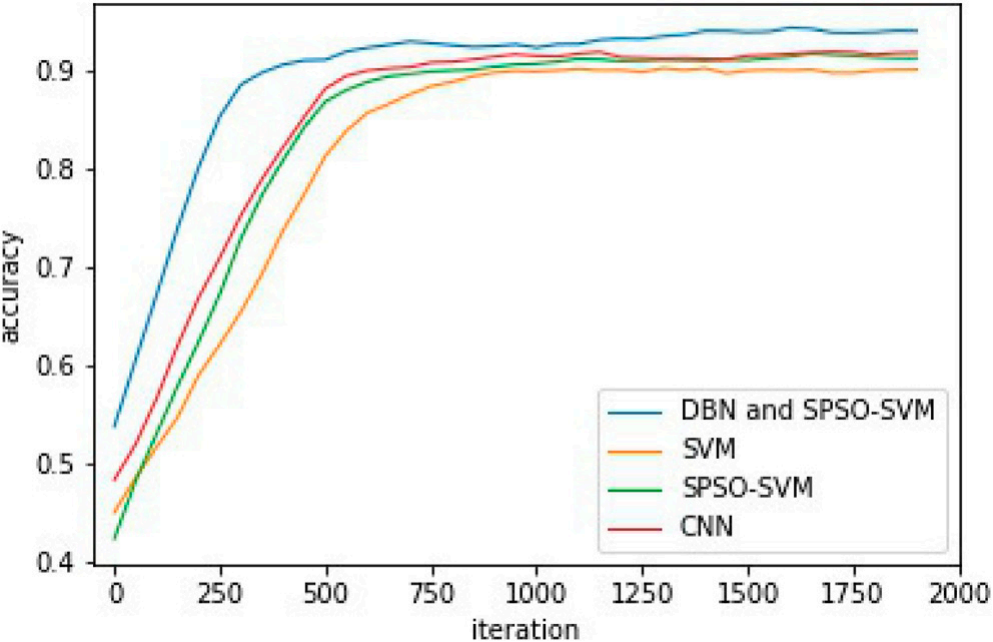
Accuracy comparison curve.

**FIGURE 10:**
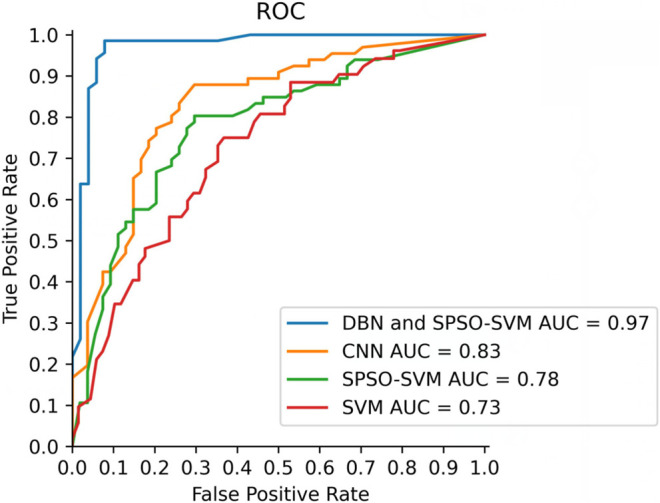
ROC curve.

For the sake of verifying the stability and performance of the DBN and SPSO-SVM models, the 10-fold cross-validation method is applied to validate the recognition results. After the model recognizes the rice diseases 30 times, the hit rate, error rate, accuracy, and the area under the receiver operating characteristic (ROC) curve (AUC) value of the model are counted. Finally, the average values of the four performance evaluation indicators are calculated. The results of cross validation are shown in Table1. Table1 shows that the hit rate and accuracy of the model are 91.37 and 94.03%, respectively, and the error recognition rate is 8.63%.The accuracy comparison curves of the four models are shown in Figure 9. It shows that the model achieves high recognition ability and guarantees a low false detection rate. The significance of the AUC value is that the closer the AUC value is to 1, the better is the recognition performance of the model. The AUC value of the recognition model proposed in this study reaches 0.97, which shows that the model has good recognition performance. The closer the ROC curve of the model is to the *Y* coordinate axis on the left and the closer to the *y* = 1 line on the top, the better is the performance of the model. The ROC curve of the model is presented in Figure 10. Figure 10 shows the ROC comparison curves of the four models. Through the comparison, it can be seen that the DBN and SPSO-SVM models used in this study have good recognition performance and are better than the other three models. It can effectively learn the implicit feature information in the data set so as to achieve better recognition effect.

**TABLE 1:** Performance parameters of the model.

Model	TPR (%)	FPR (%)	Accuracy (%)	AUC (%)
DBN and SPSO-SVM	91.37	8.63	94.03	0.97

### 5.3 Experimental Discussions

The proposed DBN and SPSO-SVM models were used to identify rice blast, sheath blight, and brown spot. Under the same experimental conditions and the same data set, support vector machine, the SPSO-SVM and convolutional neural network (CNN) models are used for comparison. The parameters of the support vector machine are set as follows: the recognition accuracy under different combinations of SVM penalty parameter C and kernel function parameter g is compared by the grid search method; when the penalty factor C of the support vector machine is 2.8 and the kernel function parameter g is 50, the recognition accuracy is the highest. The SPSO parameters are set as follows: initial population 80, inertia weight 0.9, the maximum number of iterations 300, and learning factor C1 is 1.6 and C2 is 1.8. The parameters of the CNN are set as follows: the batch size is 64 and the number of iterations is 500. Using the cross-entropy loss function, the Adam optimizer is used to minimize the loss function, and the learning rate is set to 0.001. The comparison of the results of the four models is shown in Table2. It can be seen that the DBN and SPSO-SVM models gain better results.

**TABLE 2:** Simulation results using the proposed method compared with the SVM, SPSO-SVM, and CNN.

Model	DBN and SPSO-SVM	SVM	SPSO-SVM	CNN
Accuracy rate (%)	94.03	88.92	91.02	91.43

## 6 Conclusion

This study presents a new rice disease identification method based on the DBN and SPSO-SVM. Data preprocessing, the structure design of the identification model, and the implementation technology of the DBN and SPSO-SVM have been elaborated in detail. Three features (including the color, shape, and texture) have been proposed for the DBN and SPSO-SVM method to learn in order to successfully recognize rice disease from the region of interest that is obtained by preprocessing rice disease images. Furthermore, several indices have also been proposed to verify the presented DBN and SPSO-SVM method, and demonstrate that the DBN and SPSO-SVM method achieve better recognition effect and have good anti-interference and robustness. The developed method has exhibited a better training performance, faster convergence rate, and better recognition ability than the SVM, SPSO-SVM, and CNN models.

Although the DBN and SPSO-SVM have achieved better performance in rice diseases recognition fields, there still exist some challenging problems. The first is how to find the optimal weights and bias parameters. The second is how to get high-quality rice disease sample images due to the influence of light, shooting angle, and foresight. The last one is how to improve model training effect, recognition level, and application ability.

In a future study, we plan to do the following work: investigating other optimization algorithms used to find the optimal parameters of the DBN or other machine learning models, enriching positive sample set and reducing negative sample noise interference, and extending the results in this study to some complex systems (Dong et al., 2014; Dong et al., 2015a; Dong et al., 2016a; Dong et al., 2016b; Jiang et al., 2019a; Jiang et al., 2019b; Li G. et al., 2019; Chen et al., 2021a; Chen et al., 2021b; Chen et al., 2021c; Chen et al., 2021d; Chen et al., 2021e; Huang et al., 2021; Jiang et al., 2021a; Jiang et al., 2021b; Liu et al., 2021b; Liu et al., 2021c; Liu X. et al., 2021; Zhao et al., 2021; Chen et al., 2022a; Chen et al., 2022b; Chen et al., 2022c; Sun et al., 2022; Wu et al., 2022).

## Data Availability

The raw data supporting the conclusion of this article will be made available by the authors, without undue reservation.
